# Inaccuracy of Venous Point-of-Care Glucose Measurements in Critically Ill Patients: A Cross-Sectional Study

**DOI:** 10.1371/journal.pone.0129568

**Published:** 2015-06-12

**Authors:** Adriano José Pereira, Thiago Domingos Corrêa, Francisca Pereira de Almeida, Rodrigo Octávio Deliberato, Michelle dos Santos Lobato, Nelson Akamine, Eliézer Silva, Alexandre Biasi Cavalcanti

**Affiliations:** 1 Intensive Care Unit, Hospital Israelita Albert Einstein, São Paulo, SP, Brazil; 2 School of Medicine, Universidade Federal de Lavras (UFLA), Lavras, MG, Brazil; 3 Research Institute HCor, Hospital do Coração, São Paulo, SP, Brazil; Omaha Veterans Affairs Medical Center, UNITED STATES

## Abstract

**Introduction:**

Current guidelines and consensus recommend arterial and venous samples as equally acceptable for blood glucose assessment in point-of-care devices, but there is limited evidence to support this recommendation. We evaluated the accuracy of two devices for bedside point-of-care blood glucose measurements using arterial, fingerstick and catheter venous blood samples in ICU patients, and assessed which factors could impair their accuracy.

**Methods:**

145 patients from a 41-bed adult mixed-ICU, in a tertiary care hospital were prospectively enrolled. Fingerstick, central venous (catheter) and arterial blood (indwelling catheter) samples were simultaneously collected, once per patient. Arterial measurements obtained with Precision PCx, and arterial, fingerstick and venous measurements obtained with Accu-chek Advantage II were compared to arterial central lab measurements. Agreement between point-of-care and laboratory measurements were evaluated with Bland-Altman, and multiple linear regression models were used to investigate interference of associated factors.

**Results:**

Mean difference between Accu-chek arterial samples versus central lab was 10.7 mg/dL (95% LA -21.3 to 42.7 mg/dL), and between Precision PCx versus central lab was 18.6 mg/dL (95% LA -12.6 to 49.5 mg/dL). Accu-chek fingerstick versus central lab arterial samples presented a similar bias (10.0 mg/dL) but a wider 95% LA (-31.8 to 51.8 mg/dL). Agreement between venous samples with arterial central lab was the poorest (mean bias 15.1 mg/dL; 95% LA -51.7 to 81.9). Hyperglycemia, low hematocrit, and acidosis were associated with larger differences between arterial and venous blood measurements with the two glucometers and central lab. Vasopressor administration was associated with increased error for fingerstick measurements.

**Conclusions:**

Sampling from central venous catheters should not be used for glycemic control in ICU patients. In addition, reliability of the two evaluated glucometers was insufficient. Error with Accu-chek Advantage II increases mostly with central venous samples. Hyperglycemia, lower hematocrit, acidosis, and vasopressor administration increase measurement error.

## Introduction

Although tight glucose control has failed to improve survival in critically ill patients [1], glucose control protocols are needed, since very high blood glucose levels [2], hypoglycemia [3] and glucose variability [4] may increase mortality. Point-of-care glucometers are the most commonly used devices for blood glucose measurement and guide glucose management in intensive care units (ICU) [5].

Point-of-care glucometers were developed to improve the self-management of blood glucose by diabetic patients [6], and their reliability may be insufficient for managing blood glucose in critically ill patients [5–7]. Portable glucometers are affected by environmental and therapeutic factors, such as humidity and temperature [6], ascorbic acid [8], acetaminophen and mannitol [9], and by innumerous patient-related conditions, such as hypoxemia [10,11], acidosis [10,11], hypotension [12], hematocrit [11,13] and hypoglycemia [14]. Additionally, according to the manufacturers, high triglycerides and bilirubin levels may increase the glucometers inaccuracy.

Several studies have addressed the performance of different point-of-care glucometers in critically ill patients [15]. However, those studies usually enrolled a small number of patients [16,17], were retrospective with non-concurrent blood draw from different sites [18], and/or used repeated samplings in the same subjects without accounting for the lack of independence of blood glucose values within subject [19,20].

Additionally, central venous sampling for point of care assessment of blood glucose is a common practice when arterial sampling is not promptly available, and current guidelines for the management of hyperglycemia in critically ill patients recommend either arterial or venous samples for blood glucose assessment [21]. Nevertheless, the available evidence supporting this recommendation is scarce. Only few studies have directly compared the accuracy of arterial *versus* venous samples [22], and many have reported the results including both sources indistinctly [23–25].

Our aim was to evaluate the reliability of two point-of-care devices for blood glucose measurement of samples collected simultaneously from arterial, fingerstick and venous blood of critically ill patients. Additionally, we sought to determine the influence of clinical and environmental factors on the performance of point-of-care glucometers in the intensive care unit setting.

## Methods

### Participants

We performed a cross-sectional study between March 2006 and July 2007, with prospective data collection, in critically ill patients admitted to a 41-bed intensive care unit (ICU). Inclusion criteria were admission to the ICU and periodic blood glucose measurements requested by the attending physician. There were no exclusion criteria. The local hospital ethics committee approved the protocol, and informed consent was provided by each patient or next of kin before enrollment.

### Measurements

The reference method used was the central lab measurement in serum of arterial blood glucose (dry chemistry, Vitros, Jonhson&Jonhson, New Jersey, USA) [26]. We compared whole blood glucose assessments, involving arterial measurements obtained with Precision PCx (glucose oxidase, Abbott, Illinois, USA) [27] to the arterial central lab measurement. We also compared arterial, fingerstick and venous measurements of blood glucose obtained with Accu-chek Advantage II glucometer (glucose dehydrogenase, and bioamperometry, pyrroloquinolinequinone strips; Roche, Basel, Switzerland) [28] to the arterial central lab measurement. Samples from arterial (indwelling catheter), venous (central venous catheter [CVC], double or triple lumen, 20 cm, 7Fr., 14–18 Ga.) and fingerstick blood were obtained simultaneously, and once per patient.

To avoid hemodilution and contamination of the arterial samples, 3 mL of arterial blood was collected from the indwelling arterial catheter and discarded [29]. Then, an additional 5 mL of arterial blood was drawn and immediately analyzed. One drop of the arterial blood sample was analyzed at the bedside with Precision PCx glucometer and another drop with Accu-chek Advantage II glucometer. The remaining arterial blood was sent to the central lab for blood glucose assessment. Additionally, triglycerides and total bilirubin levels were measured in the arterial blood sample. All samples were obtained using vacuum tubes containing fluoride oxalate. Right after sampling, the blood tubes were systematically sent to the central lab through a pneumatic tube transport system, to be immediately analyzed.

To avoid hemodilution by, and contamination with intravenous fluids, 5 mL of venous blood were collected from the distal lumen of the CVC and discarded prior sampling [29]. Afterwards, an additional 5 mL of venous blood was drawn and analyzed immediately with the Accu-chek Advantage II glucometer. Fingerstick blood samples were obtained from the patient’s fingertip with a lancet device, and were analyzed with the Accu-chek Advantage II glucometer. One of the investigators (F.P.A), a senior critical care nurse, supervised all procedures.

Data collected included: demographic data, the Sequential Organ Failure Assessment score (SOFA score) [30], Acute Physiology and Chronic Health Evaluation II (APACHE II score) [31], mean arterial blood pressure, peripheral body temperature, hematocrit level, arterial pH, arterial oxygen saturation, room temperature and humidity (Humidity Sensor System, Model NR 5026, Gibeck Respiration AB, Stockholm, Sweden), total bilirubin levels, triglycerides, use of vasopressors (norepinephrine and dopamine), acetaminophen, ascorbic acid, mannitol and mechanical ventilation.

### Statistical Analysis

We planned to enroll 140 patients to have adequate power to run multiple linear models with up to seven variables [32].

Categorical variables were presented as absolute and relative frequencies. Continuous variables were presented as mean and standard deviation, or as median and interquartile range (IQR).

Agreements between the point-of-care assessments (arterial measurements with Precision PCx and Accu-chek Advantage II, and fingerstick and venous measurements with Accu-chek Advantage II) and the arterial central lab measurement (reference method) were evaluated by calculating the mean differences (bias) and 95% limits of agreement according to the Bland-Altman method [33]. The 95% limits of agreement indicate the interval in which one expects that 95% of the differences between pairs of blood glucose measurements (e.g., point-of-care arterial blood versus central lab arterial blood) will lie within.

We compared the magnitude of bias between the different methods of blood glucose measurement against arterial central lab measurement [e.g., arterial blood point-of-care against arterial central lab measurement (reference) compared to fingerstick blood point-of-care against arterial central lab measurement (reference)] using paired Student’s *t* test. The variability in the differences between point-of-care measurements minus arterial central lab measurements were compared among different point-of-care methods (e.g., arterial blood point-of-care versus fingerstick point-of-care) using the F test for the homogeneity of variance.

Univariate and multivariate linear regression models were used to assess which clinical and environmental factors led to biased point-of-care measurements and to estimate the magnitude of bias. All suspect variables (mean arterial blood pressure, peripheral body temperature, hematocrit, arterial pH, arterial oxygen saturation, room temperature, room air humidity, total bilirubin levels, triglycerides, use of vasopressors, acetaminophen, ascorbic acid or mannitol) were tested in univariate models. Those with p-value ≤0.10 were included in a multiple linear regression model. A p value of <0.05 was considered significant. All analyses were performed with STATA SE 9.0 (StataCorp, Texas, USA).

## Results

One hundred forty five consecutive critically ill patients were enrolled in this study. The mean age was 60.9±20.5 years and the mean APACHE II score was 23.7±9.0. Baseline characteristics of study patients are presented in Table 1.

**Table 1:** Baseline characteristics.

Age (years)	60.9 ± 20.5
Male gender, n (%)	94 (64.8)
APACHE II score	23.7 (9.0)
SOFA score	6 (4–11)
Diagnostic category, n (%)	
Respiratory	35 (24.2)
Severe sepsis / septic shock	24 (16.6)
Solid organ transplantation	24 (16.6)
Neurologic	16 (11.7)
Postoperative	16 (11.7)
Cardiovascular	10 (6.9)
Renal and metabolic	9 (6.3)
Others	11(6.0)
Mean arterial blood pressure (mm Hg)	85 (74–97)
Body temperature (°C)	36.3 (36.0–36.9)
Hematocrit (%)	27.7 (24.6–31.5)
Arterial pH	7.39 (7.33–7.43)
Arterial oxygen saturation (%)	98 (96–100)
Room temperature (°C)	23.5 (22.8–24.4)
Room air humidity (%)	61.4 (57.3–61.7)
Total bilirubin (mg/dl)	0.9 (0.6–2.8)
Triglycerides (mg/dl)	97 (69–137)
Therapy	
Mechanical ventilation, n° (%)	90 (62.1)
Vasopressors, n (%)	57 (39.3)
Norepinephrine, n (%)	56 (38.6)
Median dose (mcg/kg/min)	0.15 (0.07–0.42)
Acetaminophen, n (%)	8 (5.5)
Ascorbic acid, n (%)	1 (0.7)
Mannitol, n (%)	2 (1.4)

Values are mean±SD, median (IQR) or n (%). APACHE II = Acute Physiology and Chronic Health Evaluation II (The score ranges from 0 to 71, with higher scores indicating more severe illness), SOFA score = Sepsis-related Organ Failure Assessment (The score ranges from 0 to 20, with higher scores indicating more severe organ failure).

Mean arterial central lab blood glucose was 146±91 mg/dl. The mean Precision PCx arterial blood glucose was 160±71 mg/dl. The mean Accu-chek Advantage II arterial, fingerstick and venous blood glucose were 151±69 mg/dl, 151±74 mg/dl and 162±90 mg/dl, respectively.

**Fig 1:**
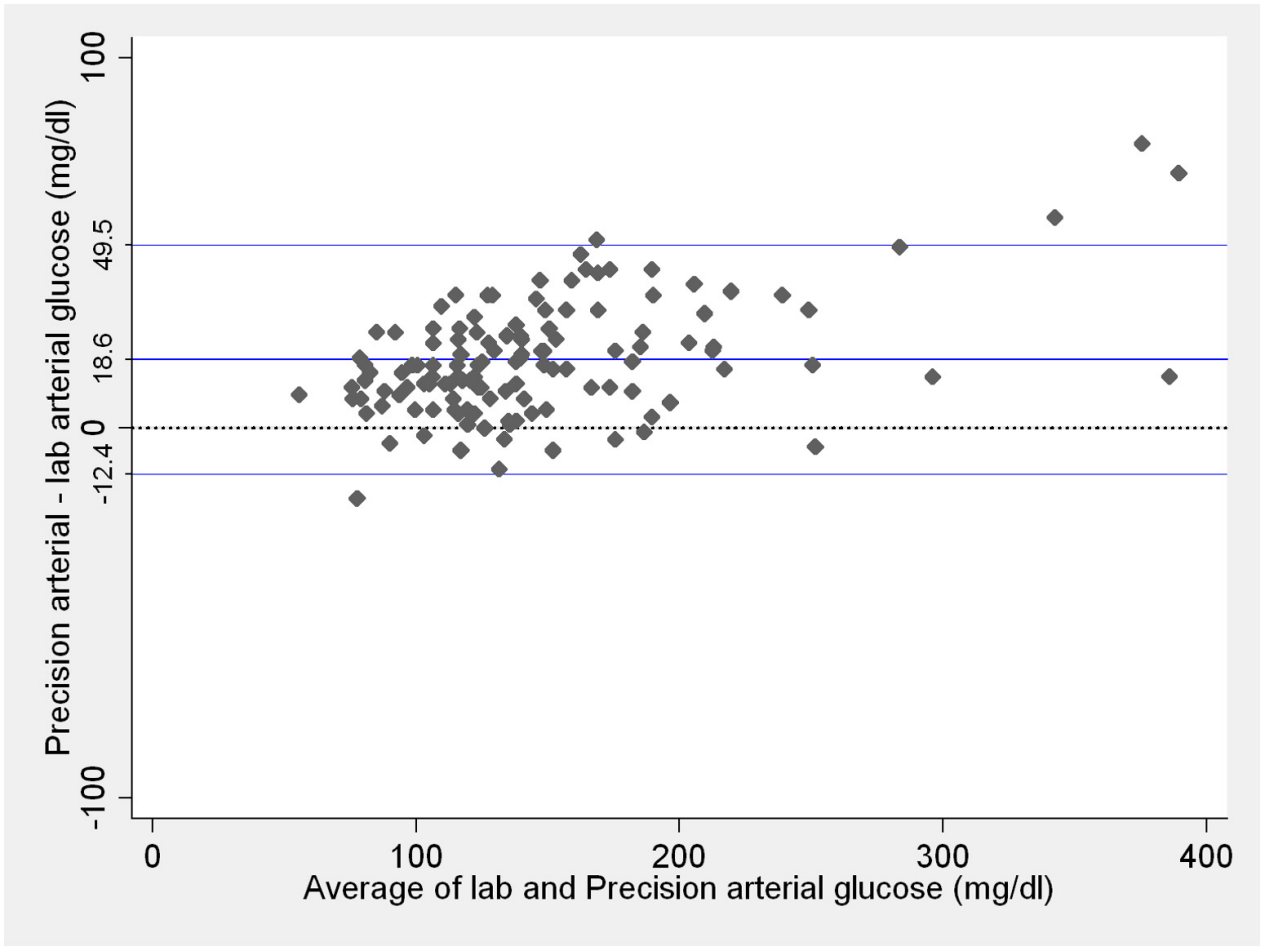
Comparison between central lab arterial and Precision PCx arterial blood glucose measurements. Blue lines represent the mean difference and 95% limits of agreement. Dotted line represents zero. Lab and Precision PCx measurements ≤ 20 mg/dL were considered as 20 mg/dL and measurements ≥ 600mg/dL considered as 600 mg/dL. These are the Precision PCx limits of detection according to the manufacturer.

**Fig 2:**
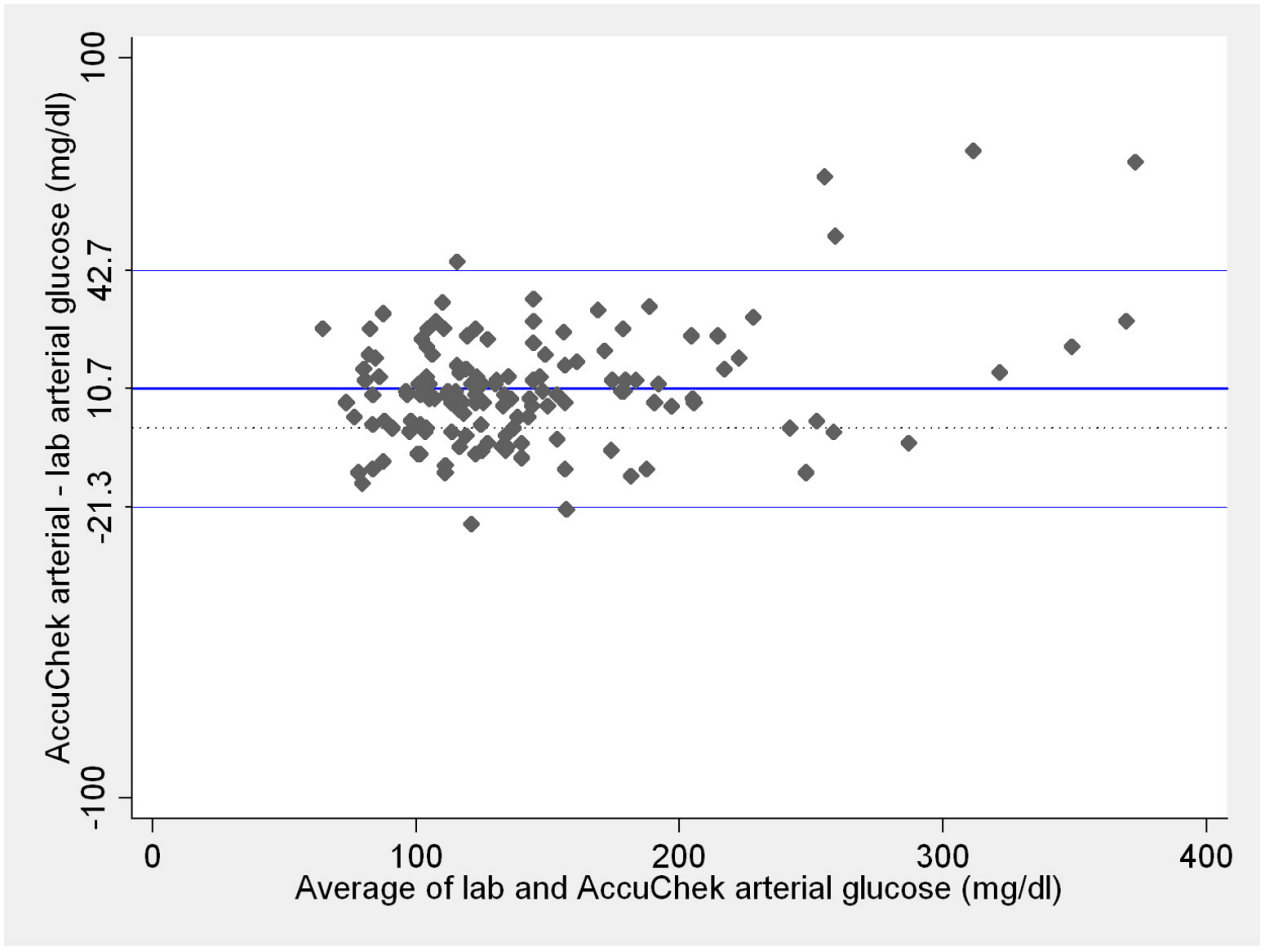
Comparison between central lab arterial and Accu-chek Advantage II arterial blood glucose measurements. Blue lines on the plot represent the mean difference and 95% limits of agreement. Dotted line represents zero. Lab and Accu-chek Advantage II measurements ≤ 10mg/dL were considered as 10 mg/dL and measurements ≥ 600 mg/dL considered as 600 mg/dL. These are the Accu-chek Advantage II limits of detection according to the manufacturer.

Mean difference (bias) between arterial blood glucose measurements performed with Precision PCx compared to arterial central lab and between arterial samples analyzed with Accu-chek Advantage II compared to arterial central lab are presented on Figs 1 and 2, respectively. Bias was greater for arterial blood glucose measurements performed with Precision PCx than measurements performed with Accu-chek Advantage II (p < 0.001), although the variability of differences between the two point-of-care methods against arterial central lab was similar (p = 0.74).

**Fig 3:**
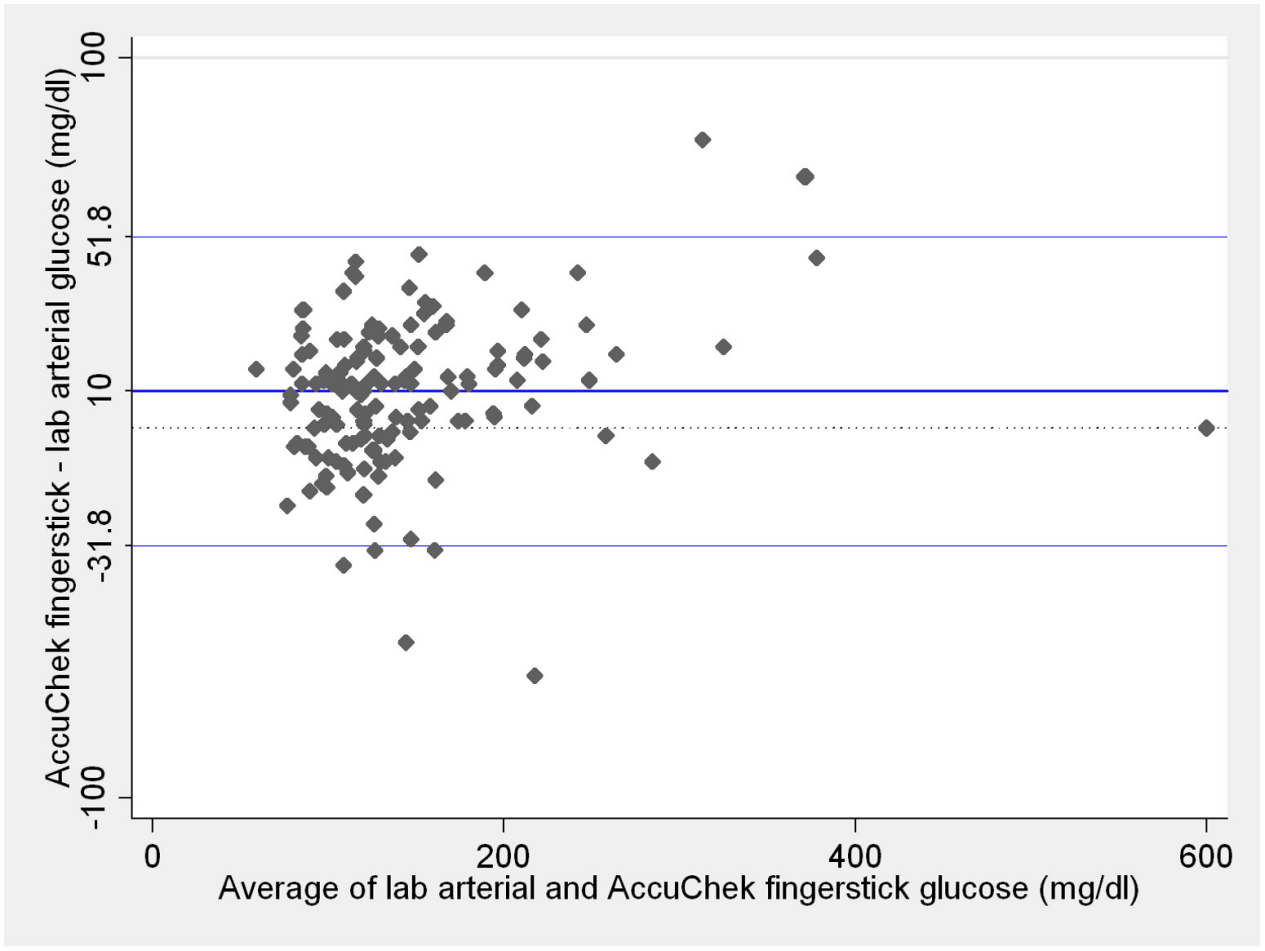
Comparison between central lab arterial and Accu-chek Advantage II fingerstick glucose measurements. Blue lines on the plot represent the mean difference and 95% limits of agreement. Dotted line represents zero. Lab and Accu-chek Advantage II measurements ≤ 10mg/dL were considered as 10 mg/dL and measurements ≥ 600 mg/dL considered as 600 mg/dL. These are the Accu-chek Advantage II limits of detection according to the manufacturer.

Mean bias between fingerstick samples analyzed with Accu-chek Advantage II versus arterial central lab measurements is presented on Fig 3. Although the mean bias of fingerstick blood glucose measurements with Accu-chekAdvantage II was similar to the bias for arterial blood glucose measurements with the same point-of-care device (p = 0.65), variability of differences was wider for fingerstick than for arterial measurements (p = 0.002).

**Fig 4:**
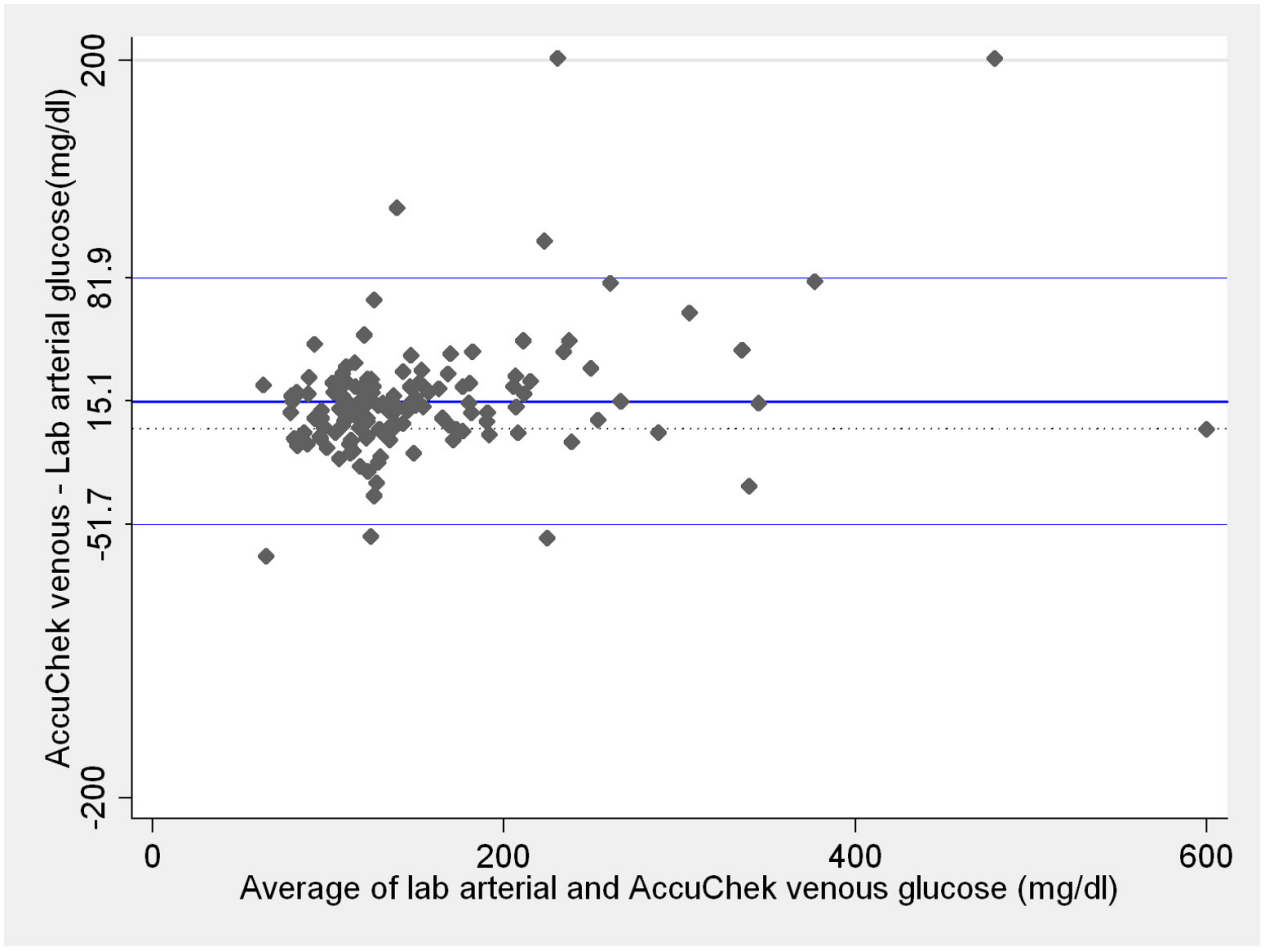
Comparison between central lab arterial and Accu-chek Advantage II venous blood glucose measurements. Blue lines on the plot represent the mean difference and 95% limits of agreement. Dotted line represents zero. Lab and Accu-chek Advantage II measurements ≤ 10mg/dL were considered as 10 mg/dL and measurements ≥ 600 mg/dL considered as 600 mg/dL. These are the Accu-chek Advantage II limits of detection according to the manufacturer.

The worst agreement was detected between venous samples analyzed with Accu-chek Advantage II versus arterial central lab measurements (Fig 4). We observed venous blood glucose values varying between 69 mg/dL lower than the central lab arterial measurement up to 474 mg/dL higher. Mean bias of venous blood glucose analyzed with Accu-chek Advantage II did not differ from mean bias of arterial blood analyzed with the same device (p = 0.17). Nevertheless, variability was wider for venous samples in comparison to arterial blood analyzed with Accu-chek Advantage II (p < 0.001).

Factors associated with measurement errors for arterial blood glucose analyzed with Precision PCx, and arterial, fingerstick and venous blood glucose analyzed with Accu-chek Advantage II are presented in Table 2. Blood glucose levels were associated with error for both point-of-care glucometers and at all sampling sites (Table 2). For instance, for each 1.0 mg/dL increase in arterial blood glucose, the mean error of arterial blood glucose measurement with Precision PCx will increase by 0.12 mg/dL (95% confidence interval: 0.08 to 0.16; p < 0.001) and by 0.10 mg/dL (95% confidence interval: 0.06 to 0.15; p < 0.001) with Accu-chek Advantage II (Table 2). Therefore, when blood glucose of a patient was 300 g/dL the mean arterial blood glucose value with Precision PCx and Accu-chek Advantage II would be 326 mg/dL and 317 mg/dL, respectively.

**Table 2:** Multiple linear regression models of clinical and environmental factors, which are independently associated with bias in blood glucose measurement with point-of-care glucometers.

Glucometer	Sampling site	Clinical and environmental factors	Measurement error (95%CI)^§^	P-value
Precision PCx	Arterial	Mean blood glucose (mg/dl)^†^	0.12 (0.08 to 0.16)	<0.001
		Hematocrit (%)	-0.89 (-1.38 to -0.40)	<0.001
Accu-chekAdvantage II	Arterial	Mean blood glucose (mg/dl)^†^	0.10 (0.06 to 0.15)	<0.001
		Hematocrit (%)	-0.74 (-1.29 to -0.19)	0.009
		Arterial pH	-34.8 (-62.8 to -6.7)	0.020
Accu-chekAdvantage II	Fingerstick	Mean blood glucose (mg/dl)^†^	0.12 (0.06 to 0.17)	<0.001
		Use of norepinephrine	-25.8 (-40.1 to -11.5)	0.001
		Hematocrit (%)	-1.04 (-1.75 to -0.32)	0.005
Accu-chekAdvantage II	Venous	Mean blood glucose (mg/dl)^†^	0.22 (0.13 to 0.30)	<0.001
		Arterial pH	-77.4 (-135.7 to -19.1)	0.010

^§^ = Measurement error represents the change in the difference of blood glucose between point-of-care glucometer and arterial central lab measurement for one unit increase in the clinical or environmental factor;

^†^ = mean blood glucose is the mean between the point-of-care and arterial central lab blood glucose values; CI = confidence interval.

Other factors which interfered with point-of-care measurements were hematocrit for arterial blood measurements performed with Precision PCx (p < 0.001) and arterial (p = 0.009) and fingerstick (p = 0.005) blood measurements performed with Accu-chek Advantage II; arterial pH for arterial (p = 0.02) and venous (p = 0.01) blood samples analyzed with Accu-chek Advantage II and finally, norepinephrine administration for fingerstick measurements performed with Accu-chek Advantage II (p = 0.001; Table 2).

**Fig 5:**
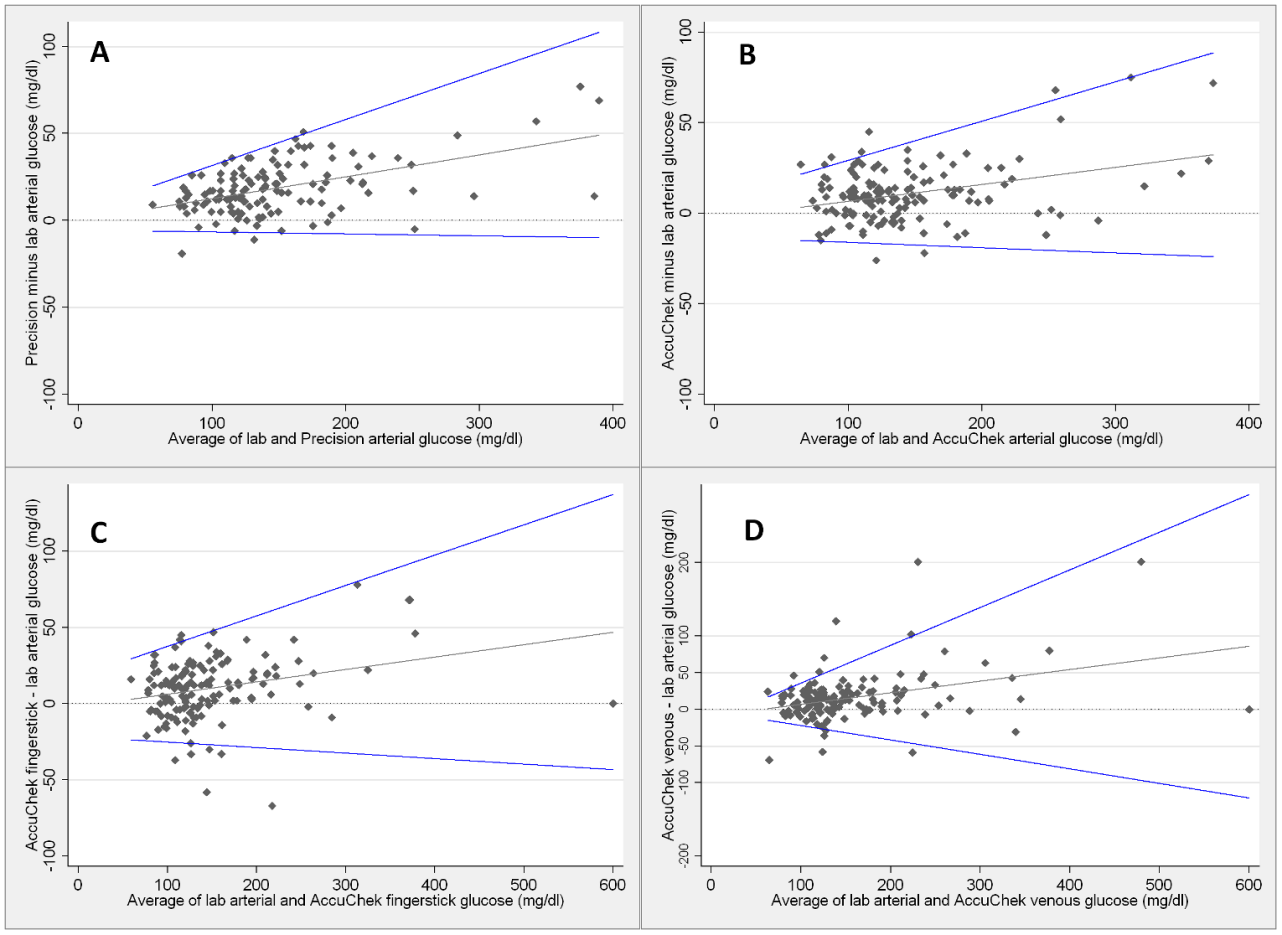
Relationship between the blood glucose values and the point-of care performance. Comparisons between arterial central lab and Precision PCx arterial (A), Accu-chek Advantage II arterial (B), fingerstick (C) and venous (D) blood glucose measurements. The gray and blue lines are the mean difference and 95% limits of agreement, respectively. Precision PCx measurements ≤ 20 mg/dL were considered as 20 mg/dL and measurements ≥ 600mg/dL considered as 600 mg/dL. These are the Precision PCx limits of detection according to the manufacturer. Accu-chek Advantage II measurements ≤ 10mg/dL were considered as 10 mg/dL and measurements ≥ 600 mg/dL considered as 600 mg/dL. These are the Accu-chek Advantage II limits of detection according to the manufacturer.

In addition to being associated with mean bias, increasing blood glucose values are also associated with larger 95% limits of agreement, implying that precision decreases during hyperglycemia (Fig 5).

## Discussion

The most intriguing result of this study was the lack of accuracy of point-of-care venous blood glucose measurements. We strictly followed a routine care to avoid contamination with I.V. fluids, discarding 5 mL of blood before the measurements. The International Federation of Clinical Chemistry recommends withdrawing a volume equal to three times the “dead space” of the catheter prior to blood sampling [29], or even less for arterial catheters [34]. Assuming that the priming volume of a double or triple lumen catheter corresponds to 0.4–0.6 cc [35], 5 mL would be more than enough to rule out sampling contamination. Analysis of catheter venous samples in portable glucometers in ICU, e.g. after laboratory sampling for other tests in patients without indwelling arterial catheters is a common issue, and raising awareness about this limitation should change clinical practice in future.

Current international guidelines consider arterial and venous samples as equivalent [21], or venous samples at least as the second option in case of arterial line unavailability [36]. However, the evidence to support that is rather limited. Several studies assumed arterial-venous glucose differences as insignificant and sampled arterial or venous sites, indistinctly [18,24,25,37], or included only venous samples as the reference to the central lab measurements [20,38,39]. One study directly compared venous to arterial blood glucose measurements in 20 patients [22]. In spite of the small sample size, authors reported a larger variability in the venous to central lab differences (95% limits of agreement: -30 to 60) compared to the arterial to central lab differences (95% limits of agreement: -5 to 35) [22]. About potential mechanisms involved, another study, in which catheter venous samples were compared to samples obtained through venipuncture, ruled out exogenous blood glucose contamination as the cause of error, and suggested that properties unique to CVC blood may interfere with Accu-Chek meter measurements [40]. Results of our study corroborate this finding.

Our study has limitations. We had limited power to estimate the effect of factors that might interfere with blood glucose measurements, in particular those with low prevalence in the population we sampled, for example use of acetaminophen or ascorbic acid. Previous data and recommendations from previous guidelines suggest that all factors we have considered may interfere in the accuracy of glucose measurements [9,41]. We have also not observed hypoglycemia in our sample. Therefore, it is possible that levels of bias and limits of agreement might be different from those we have found in lower levels of blood glucose. Finally, our findings of lower accuracy of fingerstick and venous point-of-care blood glucose measurements compared to arterial sampling are likely due to sampling site properties, but as we only assessed those samples with one type of glucometer (Accu-Check Advantage II) one can not assure those findings would be similar with Precision PCx or other devices.

There are some strengths in our work. It was a prospective study testing two devices in real conditions. We considered only one sample of each site (arterial, venous or fingerstick) per patient so the statistical assumption of independence of observations was truly held. All measurements were collect simultaneously, by a senior trained nurse, following a standardized protocol. Finally, the evaluation was not only restricted to the reliability of two glucometers and different sampling sites, but we also assessed which factors increased errors in point-of-care blood glucose measurements, being able to identify and dissect the potential sources of error during measurements, including the device itself (intrinsic error), the sample site, and the environmental and other associated factors.

## Conclusions

Due to high variability, sampling from central venous catheters should not be used for glycemic control in ICU patients. In addition, we found that the reliability of two commonly used point-of-care glucometers (Accu-chek Advantage II and Precision PCx) was insufficient in critical care, especially when using catheter venous samples. Error with Accu-chek Advantage II increases further with sampling from fingerstick blood and, most profoundly, with central venous catheter sampling. Hyperglycemia, lower hematocrit and lower pH are additional sources of error. Arterial samples seem to be the only site sufficiently accurate to be used with point-of-care glucometers.
